# The Spectrum of *NOTCH3* Variants in an Australian CADASIL Cohort

**DOI:** 10.3390/genes16111353

**Published:** 2025-11-10

**Authors:** Solomon K. Guyler, Jasmine Tsai, Neven Maksemous, Robert A. Smith, Heidi G. Sutherland, Evelyn Harvey, Andrew Duggins, Lyn R. Griffiths

**Affiliations:** 1 Genomics Research Centre, Centre for Genomics and Personalised Health, School of Biomedical Sciences, Queensland University of Technology (QUT), Brisbane, QLD 4001, Australia; solomon.guyler@hdr.qut.edu.au (S.K.G.); minhsueh.tsai@connect.qut.edu.au (J.T.); n.maksemous@qut.edu.au (N.M.); heidi.sutherland@qut.edu.au (H.G.S.); 2 Central Analytical Research Facility (CARF), Queensland University of Technology (QUT), Brisbane, QLD 4001, Australia; 3 Department of Neurology, Westmead Hospital, Westmead, NSW 2145, Australia

**Keywords:** CADASIL, compound heterozygous mutation, penetrance, severe phenotype

## Abstract

**Background** Cerebral autosomal dominant arteriopathy with subcortical infarcts and leukoencephalopathy (CADASIL) is an autosomal dominant neurological disorder caused by mutations in the *NOTCH3* gene. Disease-causing variants in *NOTCH3* are primarily missense variants altering the number of cysteine residues in the translated NOTCH3 protein. The genetic screening of the *NOTCH3* gene is currently considered the gold standard for CADASIL diagnosis. **Methods** The Genomics Research Centre has been performing diagnostic genetic testing for CADASIL since 1997. A total of 1281 patient samples suspected of having CADASIL were screened for *NOTCH3* mutations from January 1, 1997, to October 1, 2025. Genomic sequencing was performed using Sanger sequencing of selected *NOTCH3* exons or using next-generation sequencing to screen the entire *NOTCH3* gene. **Results** In total, 12.1% of patients had a cysteine-altering *NOTCH3* variant, including 49 variants in exons 2-24, and two variants in non-EGFr encoding exons. We also report the first CADASIL patient who is a compound heterozygote for two known pathogenic cysteine-altering *NOTCH3* variants, who presented with a severe early onset of stroke, migraine, and white matter changes. **Conclusions** The compound heterozygosity identified in this patient appears to be associated with an early onset of CADASIL symptoms. Our study contributes to the elucidation of the spectrum of *NOTCH3* variants associated with CADASIL. The majority of patients tested for CADASIL in this study did not contain a variant in *NOTCH3*, indicating that there are other genes or genetic variants contributing to disease in these patients.

## 1. Introduction

Cerebral autosomal dominant arteriopathy with subcortical infarcts and leukoencephalopathy (CADASIL) is a hereditary cerebral small vessel disease (CSVD), recognised as the most common genetic cause of strokes in individuals under 65 years old, and significantly contributes to neurologic deterioration in the elderly population [1,2]. The typical disease progression begins with migraines and characteristic hyperintense white matter lesions in the third to fourth decade of life, followed by ischaemic stroke and/or transient ischaemic events in the fifth decade, subcortical dementia in the sixth decade, and death by the seventh decade [3]. Magnetic resonance imaging (MRI) of affected patients displays leukoencephalopathy and subcortical infarcts typically in the external capsules and anterior pole of the temporal lobe, as well as lacunar infarcts and microhaemorrhages in some cases [4]. CADASIL is caused by heterozygous cysteine-altering variants in the epidermal growth factor-like repeat (EGFr) regions of *NOTCH3*, which result in the mispairing of cysteine residues and altered disulphide bridge formation [5]. This leads to the abnormal cleavage of the NOTCH3 extracellular domain (ECD), which aggregates in the form of granular osmiophilic material (GOM) [6]. This accumulation of is located adjacent to affected vascular smooth muscle cells (VSMCs), and is pathognomonic for CADASIL when detected in a skin biopsy [7]. It is important to note, however, that pathogenic *NOTCH3* variants are not always identified in patients with a positive CADASIL skin biopsy [8]. Conversely, not all individuals carrying a pathogenic *NOTCH3* variant exhibit GOM [7,9]. Furthermore, cysteine-sparing variants within *NOTCH3* have been detected in GOM-positive patients and, although not typically classified as CADASIL-causing, may contribute to CADASIL pathogenesis [10].

The clinical presentation of CADASIL patients is dependent upon the location of pathogenic variants. *NOTCH3* variants in EGFrs 1-6 have been associated with a 13-fold increase in the risk of stroke and vascular dementia compared to control cases, whilst variants in EGFRs 7-34 display a greater than 2-fold risk compared to healthy individuals [11]. Phenotypic variability in CADASIL is prominent; however, the age of onset and severity of symptoms often differ significantly between variants and within families [12]. While the majority of CADASIL-causing mutations are heterozygous, a small number of homozygous and compound heterozygous variants have been detected as causative of a CADASIL-like phenotype [13,14].

The Genomics Research Centre at the Queensland University of Technology has performed diagnostic genetic testing for CADASIL since 1997. This work was originally performed using Sanger sequencing of selected exons known to be hotspot regions for CADASIL variants, and was subsequently expanded to include the entirety of the *NOTCH3* gene with the advent of next-generation sequencing (NGS) [14,15]. This resulted in an improvement in diagnostic rate of CADASIL variant detection of 5%, as previously described by our laboratory [14]. The current study analyses the spectrum of *NOTCH3* variants identified in a large cohort of 1281 suspected CADASIL patients.

## 2. Materials and Methods

### 2.1. Patient Cohort

Patients were originally referred to the Genomics Research Centre NATA (National Association of Testing Authorities, Australia) accredited diagnostic laboratory by physicians in Australia and New Zealand. Patients were referred based upon a clinical suspicion of CADASIL or monogenic CSVD with a history of acute or lacunar stroke, vascular dementia, MRI features of CSVD (white matter hyperintensities, lacunes), migraine, and/or family history of stroke, vascular dementia, or small vessel disease. Ethical approval for these studies is through QUT HREC (Approval Number 1400000748). Patient results were selected from internal de-identified records from 1 January 1997 to 1 October 2025. Patients were excluded if they were family members of previously investigated probands with a confirmatory result.

### 2.2. Sanger Sequencing

Sanger sequencing was initially performed on exons 3 and 4, or an extended analysis including exons 2, 11, 18, and 19. All exons selected for analysis were based on identified mutational hotspots in *NOTCH3* (NM_000435.2). The primer sets were designed to encompass the entire exon examined and extend into the surrounding introns, resulting in amplicons of the following sizes: 193 bp (exon 2), 296 bp (exon 3), 488 bp (exon 4), 367 bp (exon 11), 258 bp (exon 18), 350 bp (exon 19) (Table S1) [16]. The methods used for *NOTCH3* Sanger sequencing were performed as previously described by Dunn et al., 2020 [14]. Samples were sequenced using the Thermo Fisher BigDye™ Terminator v3.1 Cycle Sequencing Kit (Thermo Fisher Scientific, Scoresby, VIC, Australia) and were analysed following separation on an Applied Biosystems™ 3500 Genetic Analyzer (Thermo Fisher Scientific, Scoresby, VIC, Australia).

### 2.3. Next-Generation Sequencing

NGS was conducted using an Ion AmpliSeq Custom NGS panel. The panel initially contained five genes associated with neurological disorders (*CACNA1A*, *ATP1A2*, *SCN1A*, *NOTCH3*, *TRESK*), and was expanded to include ten additional migraine or CSVD-associated genes (*TREX1*, *FOXC1*, *HTRA1*, *KCNA1*, *COL4A1*, *COL4A2*, *PRRT2*, *SCN2A*, *ATP1A3*, *GLA*) [14,17]. The patient’s peripheral blood was used to extract genomic DNA (gDNA) using the QIAamp DNA Blood Mini Kit via the automated QIAcube (QIAGEN, VIC, Australia). Library preparation was performed using the Ion AmpliSeq Library Kit 2.0 (Thermo Fisher Scientific, Scoresby, VIC, Australia). Sequencing was performed on the Ion GeneStudio S5 plus platform (Thermo Fisher Scoresby, VIC, Australia), using the Ion 550 Chip, with data analysed through the Ion Torrent suite v5.10. The generated variant call format (VCF) file for the patient sample was exported into the data analysis software, Ion Reporter v5.12 (Thermo Fisher Scientific, Scoresby, VIC, Australia) [15].

### 2.4. Bioinformatic Analysis

The results generated in the Ion Reporter utilised functional analyses such as in silico variant predictors, minor allele frequency (MAF), the type of variant (coding, non-coding), and amino acid changes (if applicable). Notable variant(s) were selected for further analysis based on predefined parameters: (i) the variant is coding or in a known region of pathogenicity; (ii) the variant is amino acid changing or predicted to alter protein product; (iii) the minor allele frequency (MAF) of the variant is less than 0.001; and (iv) multiple in silico predictors labelled the variant as pathogenic or likely pathogenic. These parameters were cross-referenced with various databases, multiple in silico variant effect predictors, and available literature for variant curation according to the American College of Medical Genetics (ACMG) guidelines [18].

## 3. Results

Diagnostic testing of *NOTCH3* was performed for 407 patients by Sanger sequencing and 874 patients by NGS of the complete *NOTCH3* gene. Fifty-one unique variants were identified in 155 patients across a total of 1281 patient samples, yielding an overall diagnostic rate of 12.1% (Table S2). Of these, 44 cases were detected by targeted Sanger sequencing of selected exons, while the remainder were identified through NGS of the entire *NOTCH3* gene. In total, 49 variants were identified as disease causing, all of which were cysteine-altering (Table S2). Two cysteine-altering variants were detected in this study and classified as variants of uncertain significance (VUS) (Table S3). These variants, p.Arg1483Cys & p.Arg2150Cys, were found in exons 25 and 33, respectively. Both variants are rare in population databases (MAF < 0.000022) but are predicted to be benign by the majority of in silico tools (Table S3). Overall, 98.7% of all detected variants were located within exons 2–24 of *NOTCH3*.

Clinical and demographic characteristics were compared between individuals carrying *NOTCH3* variants located in EGFr domains 1-6 and those in domains 7-34 (Table 1). Overall, variants were more frequently identified in females, who were on average older at the time of assessment than males. A greater number of individuals carried variants within EGFr 1-6 compared to EGFr 7-34; however, no significant differences were observed between groups in the occurrence of clinical or radiological features.

**Figure 1:**
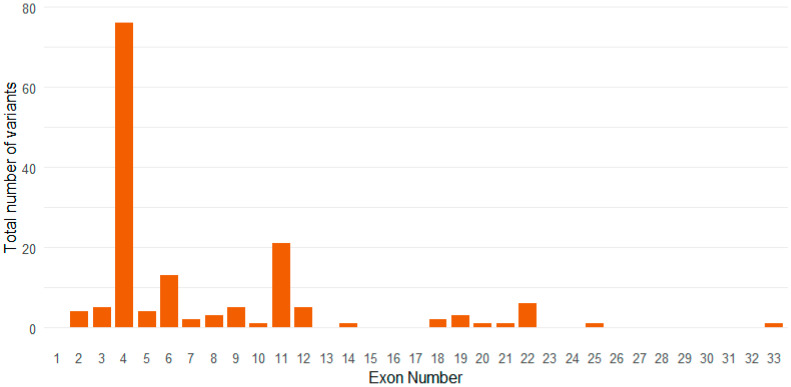
Location of *NOTCH3* Variants Stratified by Exon.

The most common exonic locations of variants in this cohort were exon 4, exon 6, and exon 11 (Figure 1). The most common disease-causing variants identified in our cohort were p.Arg141Cys (n = 25/155, 16.1%), p.Arg153Cys (n = 12/155, 7.7%), and p.Arg182Cys (n = 18/155, 11.6%), each of which was located in exon four, which is the most common location for pathogenic *NOTCH3* variants.

All variants identified in our cohort were present in a heterozygous state except for two patients. One patient was identified to possess a homozygous cysteine-altering variant, p.Arg587Cys, located in exon 11 of *NOTCH3*, in the EGFr region 15 [14]. An additional patient was determined to have two disease-causing variants, p.Arg110Cys and p.Arg1231Cys, in a compound heterozygous state. The presence of both heterozygous variants was confirmed via Sanger sequencing (Figure S1). The p.Arg110Cys variant is located in exon 3, EGFr region 2, and the p.Arg1231Cys variant is located in exon 22, EGFr region 31.

The p.Arg110Cys mutation is well established within the literature as a mechanism of CADASIL pathogenesis. In a population study of *NOTCH3* variants, only one Caucasian individual in the UK Biobank was found to harbour this variant, however this individual is likely young enough to be asymptomatic as CADASIL is a late-onset disorder [19]. The p.Arg1231Cys variant has been detected in numerous CADASIL studies as causative of disease [20,21]. Analysis of *NOTCH3* cysteine-altering variants in both the Geisinger DiscovEHR and UK Biobank cohort revealed that p.Arg1231Cys was the most common variant within the EGFr 7-34 domains [22,23,24]. In our clinical cohort, these variants have been identified individually three (p.Arg110Cys) and four (p.Arg1231Cys) times, respectively.

This 35-year-old female patient with a history of migraine had experienced two ischaemic strokes at ages 30 and 33. Brain MRI following the initial event revealed extensive T2 hyperintensities in temporal poles, suggestive of CADASIL. After the second ischaemic stroke in 2022, a skin biopsy confirmed the presence of GOM on electron microscopy. A follow-up MRI in 2023 demonstrated focal areas of increased signal intensity in the centrum semiovale and subcortical white matter, predominantly in the frontal lobes, with evidence of lacunar infarcts. Neuropsychological assessment revealed preserved cognitive and executive function but noted reductions in psychomotor processing speed and performance on complex tasks. The patient had no known cardiovascular risk factors, psychiatric history, or family history of neurological disorders or dementia. Neither parent was available for clinical evaluation or genetic testing; however, both were reportedly asymptomatic, with no history suggestive of CADASIL.

## 4. Discussion

CADASIL is the most common inherited monogenic cause of stroke and dementia worldwide. To date, 246 pathogenic variants have been described in the EGFR regions of *NOTCH3*, with 95% being identified as missense variants altering cysteine residues [3,25]. While the exact mechanisms of CADASIL pathogenesis are unknown, variants resulting in loss of function and gain of function have been identified to cause CADASIL-like phenotypes [26]. Monoallelic cysteine-altering variants have been shown to result in a toxic gain of function, where disruption in disulphide bridge formation leads to the formation of GOM around VSMCs and brain pericytes, producing toxic effects on neighbouring cells [27]. These include reduced VSMC proliferation, increased production of reactive oxygen species, impaired degradation of mutant NOTCH3 proteins, and activation of stress responses within cells [28,29].

In the current study, the majority of variants (n = 153/155; 98.7%) were located in exons 2-24 of *NOTCH3*, which encode the EGFr domains critical for disulphide bridge formation, consistent with the established disease model [30]. Of these, all variants identified as disease-causing were cysteine-altering, causing an imbalance in the number of cysteine residues, leading to disrupted disulphide bond formation. A greater number of variants were identified in early EGFr regions in this cohort, likely due to the more severe phenotype and earlier onset of disease associated with variants in these domains, as opposed to the milder severity and later onset of variants in EGFr regions 7-34 [31]. No significant differences were identified in clinical or radiological features between EGFr groups. This may be due to the later age at presentation of patients with EGFr 7-34 variants, which may still be fully penetrant, and thus result in similar clinical presentation [20]. Additionally, complete phenotype information was not available for all patients, potentially impacting the genotype-phenotype correlation in this study. This also precludes the potential for exon-specific or variant-specific analysis of phenotypes.

Two cysteine-altering variants were classified as variants of uncertain significance (VUS) in this study.

These cysteine-altering variants were identified in exons 25-33 of *NOTCH3,* which encode the extracellular domain but lie outside the EGFr-encoding regions [10,32]. Although these exons are not classically associated with CADASIL, variants within this region have been reported in individuals with a clinical CADASIL phenotype and may be impact the structural stability of the *NOTCH3* homodimer [32]. Further, loss-of-function variants in this region have been associated with CADASIL-like syndromes presenting at a much earlier age ranging from congenital onset to 17 years of age, with prominent neurodevelopmental features, including developmental delay, intellectual disability, and dysmorphic features [13]. These findings suggest that variants in exon 25-33 may contribute to CADASIL or CADASIL-like phenotypes, despite differing from the classical mutation profile.

Homozygosity and compound heterozygosity for *NOTCH3* missense variants have also been reported in the literature; however, it is exceedingly rare, with fewer than 20 cases reported to date [13,30,33]. Several reports describe homozygous missense variants in *NOTCH3*, most frequently p.Arg1231Cys in exon 22 (EGFr 31) [13]. Within our laboratory, we identified one homozygous variant, p.Arg587Cys, located in exon 11 (EGFr 15). However, the phenotype observed in this patient did not differ significantly from that seen in heterozygous cases. Overall, the majority of homozygous variants in the literature are located within EGFRs 7-34 in *NOTCH3* and are associated with a classical CADASIL phenotype, with no notable difference in age of onset or disease severity compared to individuals with the corresponding heterozygous variant [34,35,36,37,38]. Notably, only two cases have been reported with homozygous variants located in EGFR regions 1-6 (p.Arg133Cys & p.Cys183Ser), both of which were associated with an earlier and more severe CADASIL phenotype as compared to heterozygous carriers [39,40].

To our knowledge, the compound heterozygous genotype identified in the patient described here represents the first reported case involving a cysteine-altering variant within EGFr domains 1-6 in combination with another cysteine-altering variant in the EGFr regions of *NOTCH3*. All other compound heterozygote cases within the literature have been identified to affect EGFr regions 7-34 or in combination with cysteine-sparing variants [30]. Additionally, these cases have not been associated with a significantly different phenotype compared to monoallelic carriers, with a reported mean age at symptom onset of 43 years [13].

Our findings suggest that when present with another cysteine-altering variant, mutations located within the early EGFr regions of *NOTCH3* may contribute to a more severe CADASIL phenotype. This observation aligns with previously reported cases of homozygous variants identified in early EGFrs, which have been associated with increased disease severity. A high degree of inter-individual expressivity has been noted in CADASIL patients, both between variant positions and within families carrying the same variant.

It is likely that the p.Arg110Cys variant identified in this patient drives the phenotype expressed in this patient due to its position in a high-risk EGFr and known role as causative of a strong CADASIL phenotype [32]. However, the phenotype observed in the current patient may be influenced by unknown genetic interactions arising from this novel compound heterozygous genotype, or by additional genetic or environmental modifiers contributing to disease expression. However, further investigation is required, and a larger number of CADASIL patients with biallelic variants affecting early EGFrs in *NOTCH3* need to be studied before a definitive conclusion can be drawn. The inability to phase the compound heterozygous genotype identified within the patient due to an inability to perform familial segregation limits the interpretability of these variants, particularly with respect to potential inheritance of the disorder in the patient’s children.

In summary, pathogenic or likely pathogenic variants were identified in only 12.1% of patients in this study, indicating that other potential causes of disease or genetic factors need to be considered in the diagnosis of CADASIL.

## 5. Conclusions

This study defines the mutational spectrum of *NOTCH3* variants in a large clinical CADASIL cohort, demonstrating that the majority of variants occur within exons 2-24 and that cysteine-altering variants remain the primary known genetic cause of disease in these patients. The majority of patients suspected of CADASIL did not contain causal variants in *NOTCH3*, indicating that there are unknown genetic factors causing disease in these patients. The detection of two cysteine-altering missense variants in a 35-year-old female patient who presented with two ischaemic events, white matter hyperintensities, and microvascular changes on MRI highlights the role of complex inheritance in CADASIL pathogenesis. The early disease onset and severity observed in this case may reflect the cumulative deleterious effect of multiple disease-causing variants, offering further insight into the mechanisms underlying phenotypic variability in CADASIL.

## Figures and Tables

**Table 1:** Comparison of Clinical and Radiological Features in *NOTCH3* Variant Carriers by EGFr Domain.

Demographic Data	EGFr 1-6 (n = 91)	EGFr 7-34 (n = 62)	Non-EGFr Encoding Exons (n = 2)
**Mean Age (years)**	48.4	55.7	40.5
**Male, n (%)**	34 (37)	21 (34)	0
**Female, n (%)**	57 (63)	41 (66)	2 (100)
**Family history, n (%)**	31 (34)	15 (24)	0
**Stroke/TIA, n (%)**	27 (30)	19 (31)	0
**Clinical features, n (%)**	27 (30)	21 (34)	1 (50)
**MRI abnormalities, n (%)**	37 (41)	34 (55)	1 (50)
**Migraine, n (%)**	18 (20)	9 (15)	0

Values are mean or n (%). EGFr = epidermal growth factor–like repeat domain. MRI abnormalities refer to the presence of any imaging findings consistent with cerebral small vessel disease, including white matter hyperintensities, lacunes, or microbleeds, without subclassification.

## Data Availability

The original contributions presented in this study are included in the article/supplementary material. Further inquiries can be directed to the corresponding author.

## Supplementary Materials

The following supporting information can be downloaded at: https://www.mdpi.com/article/10.3390/genes16111353/s1, Figure S1: Sequence traces (reverse complement) of the two cysteine-altering variants in *NOTCH3* identified by NGS. p.Arg110Cys variant (left) and p.Arg1231Cys variant (right) with Sanger chromatogram (top) and Integrated Genomics Viewer (IGV, bottom) indicating heterozygous mutations; Table S1: Primer Sequences used for PCR amplification of *NOTCH3* exons for Sanger sequencing. Primer sequences are written in the 5′-3′ direction; Table S2: *NOTCH3* Cysteine altering variants (n = 51) identified from Sanger and next-generation sequencing according to exon, epidermal growth factor-like repeat (EGFR) region, and number of samples containing each variant, including variants previously described by Dunn et al. (2020) [14]. NOTCH3 assembly: NM_000435.3. ACMG: American College of Medical Genetics. ‘+’ = known pathogenic mechanism, ‘-‘ = not previously characterised; Table S3: In silico and Population Frequency Data for *NOTCH3* Variants of Uncertain Significance. NOTCH3 transcript: NM_000435.3. Minor allele frequencies (MAF) were obtained from the GnomAD v4.1.0 total dataset. P = Pathogenic, LP = Likely Pathogenic, VUS = Variant of Uncertain Significance.
