# Supplementary material for: The Spectrum of NOTCH3 Variants in an Australian CADASIL Cohort
Source: Genes (Basel). 2025 Nov 10;16(11):1353. doi: 10.3390/genes16111353 (PMC12651956; doi:10.3390/genes16111353)
Supplement: Supplementary file 1 [file genes-16-01353-s001.zip › genes-3932398-supplementary.pdf]

Supplementary Tables

Exon Number	Forward Primer Sequence	Reverse Primer Sequence
**2**	GAGGGGGTTTGTCACTTGG	ACACACAGGGCCCACTGGT
**3**	GCGTGTTTCTTGCCTGTCTTGTGT	AGGACAGGGTGAGTTTAGGACTGA
**4**	TAGTCGGGGGTGTGGTCAGT	TCAAACCCTAGCAGGGAA
**11**	ATTGGTCCGAGGCCTCACTT	CCATTCCCAACCCCTCTGTG
**18**	GGGGAAGCACTCAGAGTCAG	AGGTCCCCAGTAACTCCA
**19**	CCAGGTGGGTGGAGTTACTGGGG	AGCAGGAGGTACGTGCATGA

Table S1: Primer Sequences used for PCR amplification of NOTCH3 exons for Sanger sequencing. Primer sequences are written in the 5'-3' direction.

Variant	No. of Samples	Exon	EGFR Region	ACMG Classification	Known Mechanism of Pathogenesis
**p.Arg54Cys**	3	2	1	Likely Pathogenic	+
**p.Cys65Tyr**	1	2	1	Pathogenic	+
**p.Arg90Cys**	1	3	2	Pathogenic	+
**p.Arg110Cys**	4	3	2	Pathogenic	+
**p.Arg133Cys**	2	4	3	Pathogenic	+
**p.Arg141Cys**	25	4	3	Pathogenic	+
**p.Cys144Arg**	1	4	3	Likely Pathogenic	+
**p.Cys144Phe**	4	4	3	Likely Pathogenic	+
**p.Arg153Cys**	12	4	3	Pathogenic	+
**p.Arg169Cys**	6	4	4	Pathogenic	+
**p.Cys174Arg**	1	4	4	Likely Pathogenic	+
**p.Arg182Cys**	18	4	4	Pathogenic	+
**p.Cys183Arg**	2	4	4	Pathogenic	+
**p.Cys194Ser**	1	4	4	Likely Pathogenic	+
**p.Arg207Cys**	3	4	5	Likely Pathogenic	+
**p.Cys224Tyr**	1	4	5	Likely Pathogenic	+
**p.Cys233Tyr**	1	5	5	Likely Pathogenic	+
**p.Cys245Arg**	2	5	6	Pathogenic	+

**p.Cys260Arg**	1	5	6	Pathogenic	+
**p.Cys271Tyr**	2	6	6	Likely Pathogenic	+
**p.Cys291Ser**	1	6	7	Likely Pathogenic	+
**p.Gly297Cys**	1	6	7	Pathogenic	+
**p.Cys318Phe**	2	6	8	Likely Pathogenic	+
**p.Arg332Cys**	7	6	8	Pathogenic	+
**p.Gly382Cys**	2	7	9	Likely Pathogenic	+
**p.Arg421Cys**	1	8	10	Pathogenic	+
**p.Arg449Cys**	2	8	11	Likely Pathogenic	+
**p.Tyr465Cys**	1	9	11	Likely Pathogenic	+
**p.Cys473Leu**	4	9	12	Likely Pathogenic	+
**p.Cys516Phe**	1	10	13	Likely Pathogenic	+
**p.Arg544Cys**	5	11	13	Pathogenic	+
**p.Arg558Cys**	1	11	14	Pathogenic	+
**p.Cys559Tyr**	1	11	14	Likely Pathogenic	+
**p.Cys573Gly**	1	11	14	Likely Pathogenic	+
**p.Arg578Cys**	2	11	14	Pathogenic	+
**p.Cys579Arg**	1	11	14	Likely Pathogenic	+
**p.Arg587Cys**	1	11	15	Pathogenic	+
**p.Arg592Cys**	1	11	15	Pathogenic	+
**p.Cys597Trp**	5	11	15	Likely Pathogenic	+
**p.Arg607Cys**	3	11	15	Likely Pathogenic	+
**p.Arg640Cys**	5	12	16	Likely Pathogenic	+
**p.Arg717Cys**	1	14	18	VUS	+
**p.Cys977Gly**	2	18	25	Likely Pathogenic	+
**p.Arg1006Cys**	1	19	26	Pathogenic	+
**p.Arg1031Cys**	2	19	26	Pathogenic	+
**p.Tyr1106Cys**	1	20	28	Likely Pathogenic	+
**p.Cys1119Tyr**	1	21	28	Likely Pathogenic	+
**p.Cys1222Gly**	1	22	31	VUS	+
**p.Arg1231Cys**	5	22	31	Likely Pathogenic	+
**p.Arg1483Cys**	1	25	-	VUS	-
**p.Arg2150Cys**	1	33	-	VUS	-

Table S2: NOTCH3 Cysteine altering variants (n=51) identified from Sanger and next generation sequencing according to exon, epidermal growth factor-like repeat (EGFR) region, and number of samples containing each variant, including variants previously described by Dunn et al (2020). NOTCH3 assembly: NM_000435.3. ACMG: American College of Medical Genetics. '+' = known pathogenic mechanism, '-' = not previously characterised.

Amino Acid Change	MAF	CADD	REVEL	PhyloP	EGFr	ClinVar
**p.Arg717Cys**	0.00002729	25.7	0.462	8.75	18	P/LP/VUS
**p.Cys1222Gly**	0.0001729	28.6	0.524	6.09	31	LP/VUS
**p.Arg1483Cys**	0.00001164	25.7	0.328	6.83	-	-
**p.Arg2150Cys**	0.00002209	23.1	0.291	5.69	-	VUS

Table S3: In-silico and Population Frequency Data for NOTCH3 Variants of Uncertain Significance. NOTCH3 transcript: NM_000435.3. Minor allele frequencies (MAF) were obtained from the GnomAD v4.1.0 total dataset. P = Pathogenic, LP = Likely Pathogenic, VUS = Variant of Uncertain Significance.

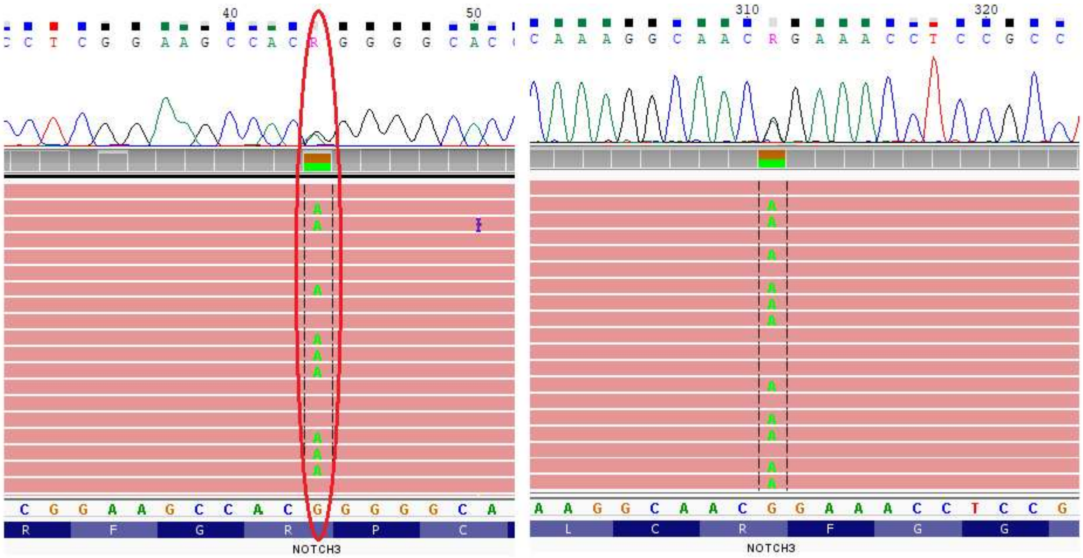

Figure S1: Sequence traces (reverse complement) of the two cysteine-altering variants in NOTCH3 identified by NGS. p.Arg110Cys variant (left) and p.Arg1231Cys variant (right) with Sanger chromatogram (top) and Integrated Genomics Viewer (IGV, bottom) indicating heterozygous mutations.
